# Aldolase B inhibits metastasis through Ten–Eleven Translocation 1 and serves as a prognostic biomarker in hepatocellular carcinoma

**DOI:** 10.1186/s12943-015-0437-7

**Published:** 2015-09-17

**Authors:** Qi-fei Tao, Sheng-xian Yuan, Fu Yang, Sen Yang, Yuan Yang, Ji-hang Yuan, Zhen-guang Wang, Qing-guo Xu, Kong-ying Lin, Jie Cai, Jian Yu, Wei-long Huang, Xiao-lei Teng, Chuan-chuan Zhou, Fang Wang, Shu-han Sun, Wei-ping Zhou

**Affiliations:** The Third Department of Hepatic Surgery, Eastern Hepatobiliary Surgery Hospital, Second Military Medical University, 225 Changhai Road, 200438 Shanghai, P.R. China; The Department of Medical Genetics, Second Military Medical University, Shanghai, China

## Abstract

**Background:**

Downregulation of Aldolase B (ALDOB) has been reported in hepatocellular carcinoma. However, its clinical significance and its role in pathogenesis of HCC remain largely unknown.

**Methods:**

We analyzed the expression of ALDOB and its clinical features in a large cohort of 313 HCC patients using tissue microarray and immunohistochemistry. Moreover, the function of stably overexpressed ALDOB in HCC cells was explored *in vitro* and *in vivo*. Gene expression microarray analysis was performed on ALDOB-overexpressing SMMC7721 cells to elucidate its mechanism of action.

**Results:**

ALDOB downregulation in HCC was significantly correlated with aggressive characteristics including absence of encapsulation, increased tumor size (>5 cm) and early recurrence. ALDOB downregulation was indicative of a shorter recurrence-free survival (RFS) and overall survival (OS) for all HCC patients and early-stage HCC patients (BCLC 0-A and TNM I stage patients). Multiple analyses revealed that ALDOB downregulation was an independent risk factor of RFS and OS. Stable expression of ALDOB in HCC cell lines reduced cell migration *in vitro* and inhibited lung metastasis, intrahepatic metastasis, and reduced circulating tumor cells *in vivo*. Mechanistically, we found that cells stably expressing ALDOB show elevated Ten–Eleven Translocation 1 (TET1) expression. Moreover, ALDOB expressing cells have higher levels of methylglyoxal than do control cells, which can upregulate TET1 expression.

**Conclusion:**

The downregulation of ALDOB could indicate a poor prognosis for HCC patients, and therefore, ALDOB might be considered a prognostic biomarker for HCC, especially at the early stage. In addition, ALDOB inhibits the invasive features of cell lines partly through TET1 expression.

**Electronic supplementary material:**

The online version of this article (doi:10.1186/s12943-015-0437-7) contains supplementary material, which is available to authorized users.

## Background

Hepatocellular carcinoma (HCC) is the fifth most common malignancy as well as the second leading cause of cancer-related deaths worldwide, and its incidence continues to rise [1, 2]. Although in the past decade, there have been improvements in surgical and medical treatments, the outcome of patients with HCC remains unsatisfactory, mainly because of frequent postsurgical recurrence and metastasis [3, 4]. Cancer classification based on biomarkers may effectively define the risk of recurrence, allowing the use of appropriate treatment for a better prognosis [5]. However, molecular markers that effectively define the risk of recurrence have not been completely explored in patients with HCC, and the underlying mechanisms responsible for metastasis remain largely unknown [4]. Therefore, more reliable biomarkers for predicting relapse and understanding the mechanisms underlying cancer metastasis need to be developed urgently [5, 6].

Aldolase B (ALDOB), also known as fructose-bisphosphate aldolase, is an important enzyme for glucose and fructose metabolism. It cleaves fructose1-phosphate to yield glyceraldehyde and dihydroxyacetonephosphate. Accumulation of fructose 1-phosphate in tissues due to a defect in ALDOB may result in hereditary fructose intolerance [7, 8]. Serum levels of ALDOB appear to be a useful measure of liver cell necrosis in both benign and malignant liver diseases [9]. Results from studies using different technologies but small sample sizes have shown that both ALDOB mRNA and protein are dramatically downregulated in HCC as compared to adjacent non-cancerous liver tissue [10–14]. However, the clinical significance of ALDOB and its role in the pathogenesis of HCC remain largely unknown.

In the current study, we found that ALDOB expression was further reduced in patients with metastasis-inclined HCC (MIH), in those with primary HCC and venous metastases (e.g., portal vein tumor thrombus) or in those with confirmed extrahepatic metastases at follow-up, compared with metastasis-averse HCC (MAH) and in those with HCC without detectable metastases. Using semi-quantitative immunohistochemistry, we explored the clinico-pathological significance of ALDOB protein in a cohort of 313 patients with HCC. Downregulation of ALDOB was associated with multiple malignant characteristics of HCC. Multiple analyses revealed that the downregulation of ALDOB was an independent risk factor for recurrence-free survival (RFS) and overall survival (OS). Moreover, ALDOB downregulation was consistently indicative of a shorter RFS and OS in BCLC 0-A and TNM I stage patients. Furthermore, we generated stable ALDOB expressing cell lines to explore the role of ALDOB in HCC. We found that overexpression of ALDOB could inhibit cell migration and reduce metastasis *in vitro* and *in vivo*. A gene microarray study on an ALDOB stably expressing cell line and its control showed that overexpression of ALDOB induced a high level of Ten–Eleven Translocation 1 (TET1) expression. We also found that ALDOB stably expressing cells had a high level of methylglyoxal (MG), which contributes to TET1expression.

## Results

### ALDOB expression was further reduced in metastasis-inclined HCC and in cases of portal vein tumor thrombus

**Fig. 1:**
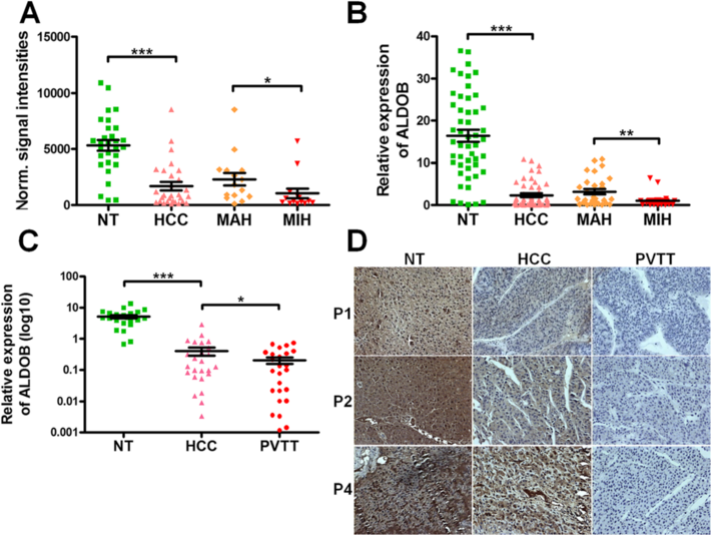
ALDOB were further reduced in metastasis-inclined HCC and in cases of tumor thrombus. **a** Normalized signal intensities of ALDOB in HCC and paired non-tumor tissue (NT) in microarray analysis. The HCC cases included 14 MIH and 15 MAH. **b** Relative expression of ALDOB mRNA in 50 cases of HCC, including 20 MIH and 30 MAH. The expression of ALDOB was analyzed by RT-PCR and normalized to β-actin. **c** The relative Expression of ALDOB mRNA in 25 HCCs with PVTT. The expression of ALDOB was analyzed by RT-PCR and normalized to β-actin. **d** Representative IHC stains of ALDOB in matched NT/HCC/PVTT

Our previously acquired microarray data (GSE54238) showed downregulation of ALDOB in HCC as compared to normal liver or paired non-tumor tissue. In this study, we found that its expression was further reduced in metastasis-inclined HCC (MIH) as compared to metastasis-averse HCC (MAH) (Fig. 1a). This indicates that ALDOB might play a role in development and progression of HCC. To validate this result, we performed qRT-PCR analysis of 50 HCC specimens (including 20 MIH and 30 MAH) and paired non-tumor tissues (NT). We found that the mRNA level of ALDOB was significantly downregulated in most HCC tissues (Fig. 1b). In agreement with the microarray results, ALDOB mRNA expression was further reduced in MIH (Fig. 1b). Immunohistochemical analysis showed that the protein level of ALDOB was also downregulated in 68.06 % (196/288) patients with HCC (Additional file 1: Figure S1A and B). Portal vein tumor thrombus (PVTT) is the primary route of metastasis in patients with HCC, and is strongly correlated with poor prognosis. We examined the expression of ALDOB in 25 cases of PVTT, matched primary tumors and non-tumor tissues. We found that the levels of ALDOB mRNA and protein were significantly lower in cases of PVTT than in primary tumors, as shown in Fig. 1c and d. These results suggest that ALDOB may play a role in suppressing HCC metastasis.

### ALDOB downregulation is associated with aggressive clinico-pathological traits

We then performed IHC on the TMA that included 313 HCC specimens to explore the clinical significance of ALDOB. The expression was recorded by the evaluation of the staining intensity of the positive cells (0 = none; 1 = +weak; 2 = ++ intermediate, 3 = +++ strong), as described above. Representative TMA core staining and scoring is shown in Additional file 1: Figure S1 C and D. Next, we examined the relationship between ALDOB expression and the clinico-pathological characteristics of the 313 patients with HCC (Additional file 2: Table S1). This analysis revealed that ALDOB downregulation was significantly correlated with female gender (*P* = 0.016), tumor size >5 cm (*P* = 0.006), absence of encapsulation (*P* = 0.002) and early recurrence (*P* = 0.002). The result also showed that downregulation of ALDOB was associated with multiple aggressive characteristics of HCC.

### ALDOB downregulation serves as a prognostic factor for patients with HCC

**Fig. 2:**
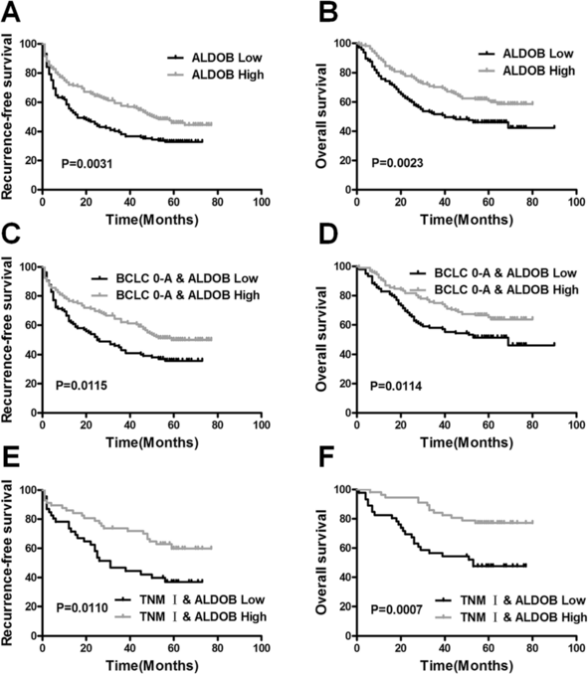
The downregulation of ALDOB serves as prognostic marker in patients with HCC. **a** and **b** The low ALDOB expression group had a shorter RFS and a shorter OS than the high ALDOB expression group. The prognostic significance was assessed by Kaplan–Meier survival estimates and a log-rank test. ALDOB downregulation also indicated worse RFS and OS patients with in BCLC0-A stage (**c** and **d**) or TNM I stage tumors (**e** and **f**)

We further sought to determine whether downregulation of ALDOB was correlated with the prognosis of patients with HCC after hepatectomy. Kaplan–Meier analysis was used to compare the subgroups with (*n* = 145) and without (*n* = 168) ALDOB downregulation (Fig. 2a and b). Remarkably, patients showing downregulation of ALDOB expression had worse RFS and OS (*P* = 0.0031, *P* = 0.0023, respectively). The 1- and 3 year cumulative recurrence rates were significantly higher in patients with ALDOB downregulation than in those without ALDOB downregulation (44.3 and 62.0 % versus 28.0 and 42.4 %; *P* = 0.0031). In addition, downregulation of ALDOB inversely correlated with 1-and 3 year survival rates (75.9 and 51.7 % versus 87.5 and 70.2 %; *P* = 0.0023). A univariate analysis revealed that HBs and HBe antigens, cirrhosis, tumor size, micro-vascular invasion, macro-vascular invasion, encapsulation, BCLC stage, TNM stage, and ALDOB expression level were significantly correlated with RFS. Additionally, these parameters also correlated significantly with OS (Additional file 2: Table S2). All significant clinico-pathological parameters were then used for multiple analyses. A Cox proportional hazards model showed that ALDOB expression level was an independent risk factor for RFS (hazard ratio [HR] 1.351, 95 % confidence interval [CI] 1.006–1.815, *P* = 0.045) and OS (hazard ratio [HR] 1.437, 95 % confidence interval[CI] 1.030-2.003, *P* = 0.033) of patients with HCC after curative hepatectomy (Table 1).

**Table 1:** Multivariate analysis of several variables for OS and RFS

Variable^a^	Recurrence-Free Survival		Overall Survival	
	Hazardratio(95 % CI)	*P*-value	Hazardratio(95 % CI)	*P*-value
HBs antigen, positive	2.046(1.221–3.430)	0.007^*^	2.173(1.171–4.031)	0.014^*^
HBe antigen, positive	1.526(1.093–2.131)	0.013^*^	1.517(1.050–2.193)	0.027^*^
Liver cirrhosis, Present	1.485(1.070–2.061)	0.018^*^	--	--
Tumor size(cm),>5	1.537(1.143–2.066)	0.004^*^	1.713(1.225–2.395)	0.002^*^
No. tumor, Multiple	--	--	--	N.S.
Differentiation, III + IV	--	N.S.	--	N.S.
Micro-vascular invasion, Present	--	N.S.	--	N.S.
Macro-vascular invasion, Present	--	N.S.	--	N.S.
Encapsulation, Absent	1.527(1.113–2.097)	0.009^*^	1.634(1.135–2.351)	0.008^*^
TNM stage, II + III	--	N.S.	--	N.S.
BCLC stage, B + C	1.775(1.288–2.444)	0.001^*^	1.917(1.351–2.721)	0.000^*^
ALDOB expression, Low	1.351(1.006–1.815)	0.045^*^	1.437(1.030–2.003)	0.033^*^

N.S., not significant

*P < 0.05 by Cox proportional hazards regression model

^a^Variables were adopted for their prognostic significance by univariate analysis

Tumor staging systems based on clinico-pathological characteristics (e.g., BCLC and TNM staging systems) can be used to predict outcomes of patients with HCC. However, correlation between the tumor stage and actual outcome is not always observed. Some patients with early stage HCC show poor prognosis, presenting clinicians with a major challenge in prognostic prediction for these patients. We therefore investigated the prognostic value of ALDOB in early stage HCC. All 313 patients were stratified according to the BCLC and TNM staging systems. Kaplan–Meier plots of HCCs of different BCLC and TNM stages are shown in Fig. 2d-g. Of the 237 patients at BCLC0-A stage, 105 had tumors with low expression levels of ALDOB. Patients with ALDOB downregulation had a poorer surgical prognosis and a worse RFS and OS than the others (37.3 and 57.0 % versus 23.5 and 37.9 % for the 1 and 3 year cumulative recurrence rates, respectively; *P* = 0.0115; 82.9 and 58.1 % versus 90.2 and 75.0 % for the 1 and 3 year overall survival rates respectively; *P* = 0.0114) (Fig. 2c and d). Similarly, of the 103 patients with TNM I stage HCC, 46 patients with ALDOB downregulation had a poorer surgical prognosis as compared to the remaining 57 patients (26.3 and 53.3 % versus14.0 and 26.3 % for the 1 and 3 year cumulative recurrence rates respectively; *P* = 0.0110; 82.6 and 56.5 % versus 96.5 and 86.0 % for the 1 and 3 year survival rates respectively; *P* = 0.0007) (Fig. 2e and f).

Thus, these data indicate that ALDOB could be used as a prognostic biomarker and a complement for prognostic algorithms (i.e., BCLC and TNM staging systems), which are based on clinico-pathological characteristics of patients with HCC.

### ALDOB inhibits HCC metastasis *in vitro* and *in vivo*

**Fig. 3:**
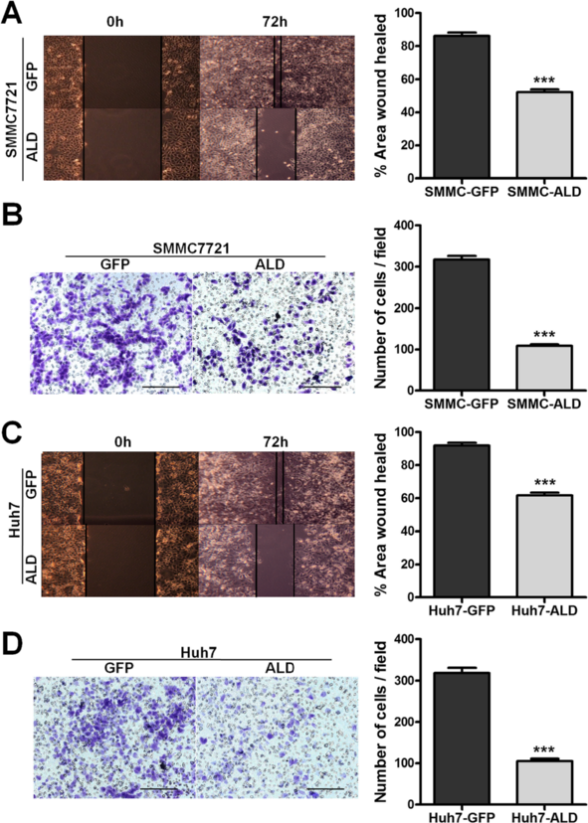
ALDOB inhibits HCC migration *in vitro*. The scratch wound healing assays and the Transwell migration assays showed that the overexpression of ALDOB inhibits the migratory properties of the HCC-derived cell lines SMMC7721 (**a** and **b**) and Huh7 (**c** and **d**). The representative results and statistical analysis are shown. Scale bars, 200 μM

To explore the biological functions of ALDOB in HCC *in vitro*, we acquired three HCC cell lines that stably expressed ALDOB: SMMC-7721, Huh7, and LM6 (named SMMC-ALD, Huh7-ALD, and LM6-ALD, respectively). The expression of ALDOB was validated by western blot (Additional file 3: Figure S2 C). In the wound healing migration assay, microscopic examination at 0 and 48 h showed significant delay in the migration of SMMC-ALD as compared to the negative control (Fig. 3a). A Transwell migration assay also revealed decreased invasiveness in SMMC-ALD cells (Fig. 3b). Similar results were observed using the other two cell lines (Fig. 3c and d, Additional file 4: Figure S3 A-D).

**Fig. 4:**
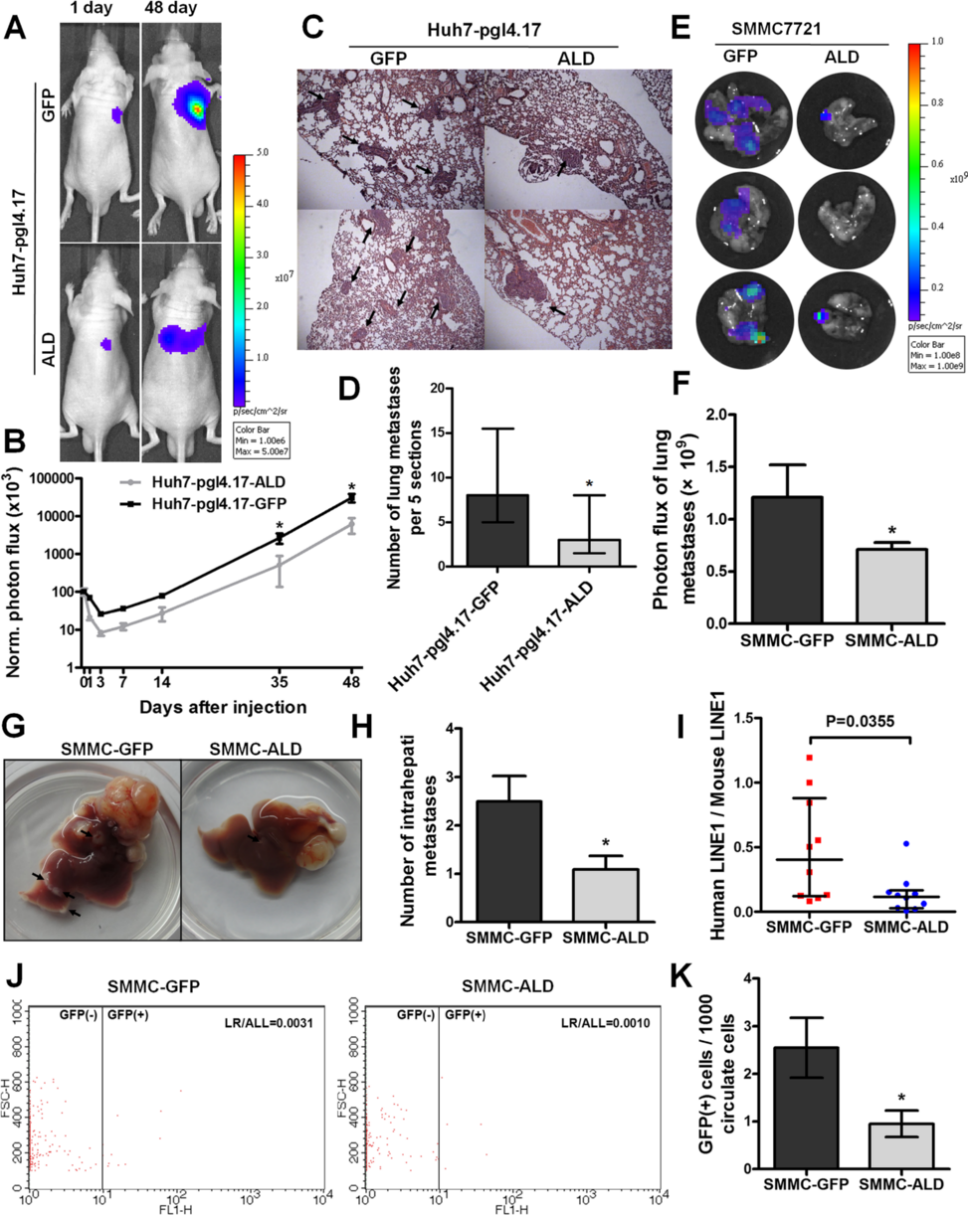
ALDOB inhibits HCC metastasis*in vivo*. **a** Images of lung metastases that developed in Huh7-pgl4.17 lateral tail vein injection models by the IVIS Imaging System. Representative luciferase signals captured in each group at the initial injection and 48 days after the injection of the cells are shown. The statistical analysis is shown in (**b**). **c** Representative H&E stained images of lung metastatic loci from each group in A. The statistical analysis is shown in (**d**). **e** Images of lung metastases that developed in SMMC7721 lateral tail vein injection models by the IVIS Imaging System. Representative GFP signals were captured in each group 49 days after the mice were euthanized. The statistical analysis is shown in (**f**). **g** Representative image of intrahepatic metastases 8 weeks after orthotopic xenograft transplantation of the indicated SMMC7721 cell clones in nude mice. The number of intrahepatic metastases in each group is shown in (**h**). **i** The relative levels of human LINE1 DNA were analyzed by qPCR of genomic DNA from whole-blood samples 48 days after orthotopic xenograft transplantation of the indicated SMMC-7721 cell clones; expression levels were normalized to mouse LINE1 DNA. **j**The number of GFP-labeled CTCs was examined by flow cytometry from whole-blood samples. The statistical analysis is shown in (**k**)

To verify the function of ALDOB *in vivo*, we injected SMMC-ALD and Huh7-PGL-ALD cells into the lateral tail vein of mice to establish a lung metastasis model. Since Huh7-PGL-ALD and the corresponding control cells Huh7-PGL-GFP expressed firefly luciferase, the process of lung metastasis was dynamically monitored every two or three weeks using an *in vivo* imaging system. The photon flux curves indicated that ALDOB expression inhibited lung metastasis in the lungs (Fig. 4a and b). After 8 weeks, the lungs were dissected and stained with hematoxylin and eosin (H&E). We found that lungs from the control group showed significant and frequent micrometastases (Fig. 4c and d). Since the SMMC-ALD cells and the corresponding control cells expressed equal levels of green fluorescent protein (GFP), we detected metastases in lungs using GFP signals, 8 weeks after injecting the cells. Weaker signals were observed in lungs of mice injected with SMMC-ALD, indicating that ALDOB also inhibits lung metastasis of SMMC7721 cells (Fig. 4e and f).

Furthermore, we injected SMMC-ALD and SMMC-GFP cells subcutaneously into nude mice. Weekly tumor volume measurements showed the ALDOB overexpression group generated less number of tumors (5/8) compares to the controls (8/8) (Additional file 3: Figure S2 F). We then transplanted tumor tissues into the liver to establish an orthotopic mouse model. We assessed tumor intrahepatic metastases 8 weeks after transplantation and observed fewer intrahepatic metastases in the SMMC-ALD group (Fig. 4g and h). As both the control and ALDOB overexpressing cells were labeled with GFP, we used flow cytometry to examine the numbers of circulating tumor cells (CTCs) from whole-blood samples obtained from the orthotopic model. Overexpression of ALDOB decreased the number of CTCs (Fig. 4j and k). We validated this result by examining human LINE1 DNA, another indicator of CTCs, in the whole-blood samples and calculated its ratio to mouse LINE1 DNA to reflect the number of CTCs. Consistent with the above results, mice transplanted with the SMMC-ALD tumors showed less human LINE1 DNA (Fig. 4i). These data demonstrate that ALDOB could inhibit intrahepatic metastasis and the spread of the tumor into the circulation system.

### ALDOB inhibits cell migration partly by elevating TET1 expression

To explore the molecular mechanism of ALDOB in the metastasis of HCC cells, we first examined the effects of ALDOB overexpression on glycolysis because of its function in the glycolytic pathway. We measured the expression levels of other glycolysis genes, in addition to the amount of glucose and lactic acid, the initial and final products of glycolysis in the ALDOB-expressing cells. However, no significant aberrations were detected in the expression of the tested genes and metabolic products of glycolysis (Additional file 5: Figure S4 A-C).

**Fig. 5:**
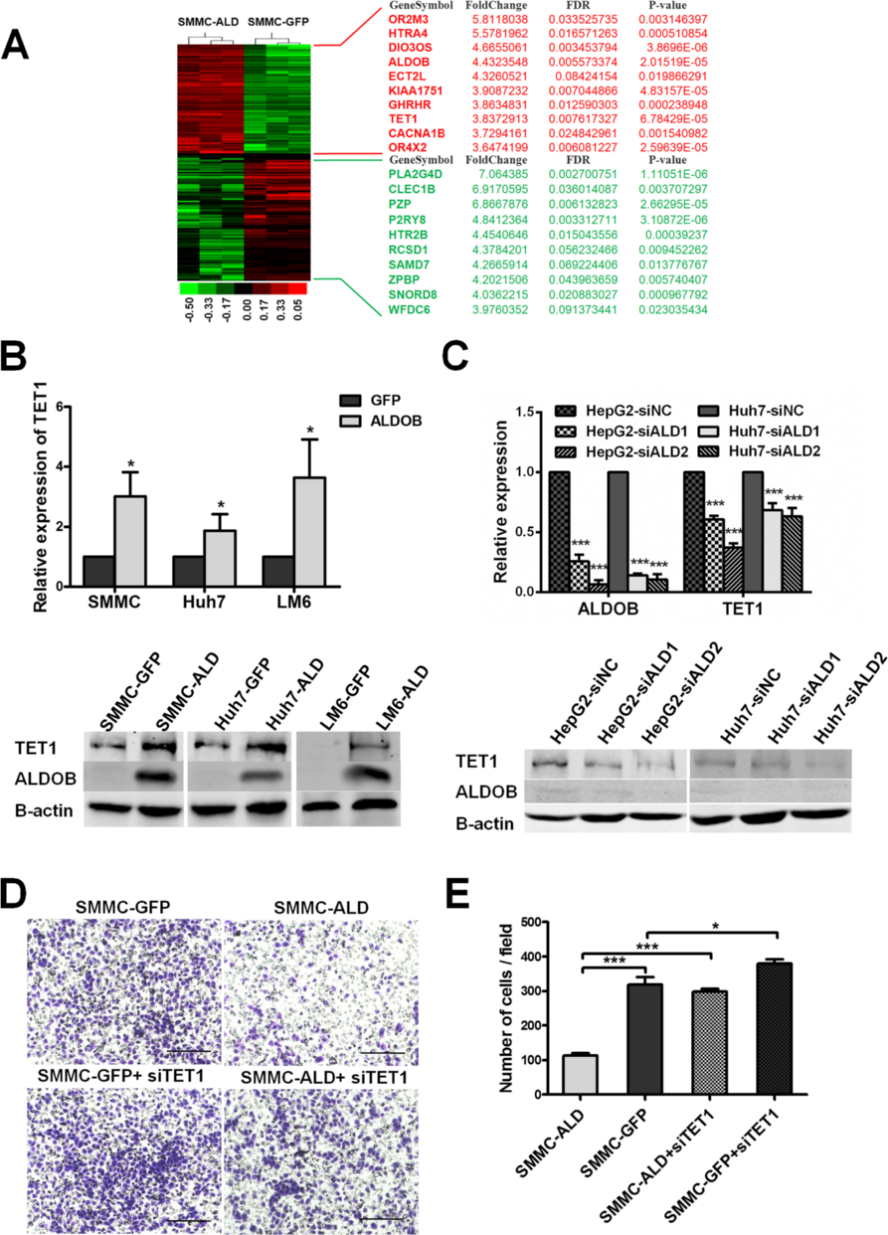
ALDOB inhibits cell migration partly though the elevation of TET1. **a** Differentially expressed genes in SMMC7721-ALDOB cells compared with control cells. The top 10 up/down-regulated genes are listed on the right. **b** Relative expression of TET1 in ALDOB stably expressing cells and paired controls. The expression of TET1 was analyzed by RT-PCR and western blot and was normalized to β-actin. **c** Relative expression of TET1 in HepG2 and Huh7 cells with siALDOB and in paired controls. The expression of TET1 was analyzed by RT-PCR and western blot and was normalized to β-actin. **d** Transwell migration assays showed that siTET1 could partly relieve the inhibitory effect induced by ALDOB. Scale bars, 200 μM. The statistical analysis is shown in (**e**)

We then analyzed the resultant expression profile of SMMC-ALD cells using a gene microarray platform. Differentially expressed genes were then included in functional classification analyses. A total of 167 genes were significantly upregulated, and 196 genes were downregulated (>2-fold, ALDOB/GFP) (Fig. 5a). GO analyses and pathway analyses showed that several pathways were modulated when ALDOB was upregulated (Additional file 6: Figure S5 A-D).

In addition, we found that expression of TET1, one of the top ten elevated genes in SMMC-ALD cells, was dramatically induced by ALDOB (Fig. 5a). Considering the role of TET1 in metastasis and cell adherence [15–17], we first validated its upregulation in cells that stably expressed ALDOB, as well as its downregulation in ALDOB-knockdown cells (Fig. 5b and c). We also found the HOXA9, a known target gene of TET1, upregulated in the ALDOB expressing cells (Additional file 5: Figure S4 F). Next, we examined the migration potential of SMMC-ALDOB cells with/without TET1 knockdown using the Transwell migration assay. We observed that when TET1 was knocked down by siRNA, the inhibitory effects induced by ALDOB were alleviated (Fig. 5d and e). This indicates that ALDOB inhibits metastasis at least partly through elevation of TET1. Some reports have shown that ALDOB increases the levels of methylglyoxal (MG) in endothelial cells [18, 19] and that MG might decrease migration of HCC cells [20]. In addition, MG can also cause elevation of TET1 expression in human lens endothelial cells (HLECs) [21]. Therefore, we examined the concentration of MG in in control cells and cell lines that expressed ALDOB. Interestingly, we found that MG was higher in ALDOB-expressing cells than in the control cell lines (Additional file 5: Figure S4 D). When we added increasing concentrations of MG (0/0.312/0.625/1.25/2.5/5 mM) to HCC cell lines, we observed notable dose-dependent upregulation of TET1, as compared to the controls (Additional file 5: Figure S4 E). These data indicate that the upregulation of TET1 expression by ALDOB might be mediated through elevation of MG levels.

## Discussion

Despite recent improvements in surgical and medical treatment strategies, prognosis of patients with HCC still remains unsatisfactory due to frequent recurrence and metastasis [3, 4]. However, the mechanisms underlying metastasis remain largely unknown, and the molecular markers that effectively define the risk of recurrence in patients with HCC have not been greatly explored. Therefore, reliable biomarkers are needed to predict relapse and outcome of patients for their optimal medical management. Moreover, additional biomarkers that play a role in HCC are needed to explore novel therapeutic targets.

Increased glycolysis, a hallmark of malignant cancers, correlates with invasive potential and poor prognosis [22]. Many glycolysis-related genes are abnormally expressed in HCC tissues, and some of these genes are thought to be involved in carcinogenesis of hepatocytes; moreover, they influence the prognosis of patients with HCC [23, 24]. ALDOB, an important glycolytic enzyme, is a well-known specific marker of hepatic differentiation and is expressed abundantly in the adult liver [25, 26]. ALDOB expression is frequently decreased in liver cancer [10–14, 24]. However, its clinical significance and role in HCC pathogenesis are largely unknown. Our previous microarray data demonstrated the dramatic downregulation of this gene in HCC tissues compared with paired non-tumor tissues. More interestingly, the expression of ALDOB is further reduced in MIH. This suggests that ALDOB could be used as a biomarker for aggressive HCC and that it might be involved in the progression of HCC.

Hence, in this study, we explored the clinical significance and role of ALDOB in HCC pathogenesis. We measured the ALDOB protein levels in a large cohort of 313 patients and correlated them with the clinico-pathological features of the patients. ALDOB downregulation correlated with female gender (*P* = 0.016), tumor size >5 cm (*P* = 0.006), absence of encapsulation (*P* = 0.002) and early recurrence (*P* = 0.002). A Kaplan–Meier analysis showed that patients with lower ALDOB expression had higher recurrence rates (*P* = 0.0031) and shorter survival times after curative resection (*P* = 0.0023). Moreover, a multivariate analysis revealed that the ALDOB level was an independent risk factor for both RFS and OS.

Prognostic algorithms (the BCLC and the TNM staging systems) based on multiple clinico-pathological characteristics have better prognostic functions than algorithms based on a single clinico-pathologic factor. However, even patients with tumors of the same stage can have different outcomes [27]. Based on the current staging systems, patients with early stage tumors are thought to be at a lower risk for recurrence. However, some of these patients may still show poor prognosis in clinical practice. Therefore, predicting the prognosis of these patients is a major challenge for attending clinicians. Considering the downregulation of ALDOB in patients with poor prognosis, we explored its prognostic value in tumors showing different stages according to the BCLC and the TNM staging systems. We first stratified all 313 patients according to the BCLC and TNM staging systems. The patient distribution and 3 year survival rates in this series were similar to those of previous reports [28]. Intriguingly, ALDOB downregulation was consistently indicative of a poor RFS and a poor OS in patients with BCLC 0-A and TNM I stage tumors. Thus, the findings of the present study suggest that measurement of ALDOB protein levels could identify a worse prognosis among patients in early stages of HCC. The prognostic value of ALDOB may be valuable to clinicians for distinguishing early stage patients with a high risk of recurrence. In such cases, close follow-up and appropriate adjuvant therapies should be recommended to prolong survival.

ALDOB overexpression also inhibited the motility and metastatic potential of HCC cells both *in vitro* and *in vivo*. In addition, we explored the underlying molecular mechanism of action of ALDOB. As no significant aberration was observed in the expression of glycolysis-related genes and initial and final metabolic products of glycolysis, we speculated that ALDOB might not affect glycolysis. This might due to: First, ALDOA, another enzyme has similar function, predominantly expressing in cell lines than ALDOB (Additional file 3: Figure S2 A and B). Second, ALDOB has a 10 fold lower Km for glyceraldehyde 3-phosphate (G3P) and dihydroxyacetone phosphate (DHAP) than ALDOA [29], which means ALDOB having the less ability of conversion fructose-1,6-bisphosphate F-1,6-BP to G3P and DHAP in glycolysis. Third, the ALDs are not the key enzyme in controlling glycolysis rate. It is reported that ALDOA knocking down does not affect the glycolytic flux and intracellular ATP concentration [30]. However, we found that the MG level was elevated in ALDOB-expressing cells, which is primarily generated from G3P and DHAP through non-enzymatic fragmentation of triosephosphates [31]. This might rely on the ability of ALDOB in cleaving the fructose-1-phosphate (F-1-P) into G3P and DHAP [32], and this might be the reason why is ALDOB but not ALDOA affect the MG level in endothelial cells [18, 19]. We validated the relationship of MG level and ALDOB expression in HCC cells. However, the influences of ALDOB in metabolism still need further exploration. MG has been reported to inhibit cell growth in several types of tumors. In HCC cell lines, MG is thought to influence cell adhesion and inhibit cell migration. However, since it is difficult to eliminate MG, whether ALDOB inhibits tumor migration mainly through MG is still unclear.

We therefore analyzed the resultant expression profile upon ALDOB overexpression. We observed that increased levels of ALDOB elevated the expression levels of TET1. TET1 belongs to the ten-eleven translocation (TET) family that promotes DNA demethylation by catalyzing 5-methylcytosine (5mC) to 5-hydroxymethylcytosine (5hmC), and to a lesser extent, 5-formylcytosine or 5-carboxylcytosine [33]. TET1 is suppressed in several solid tumors including HCC and is thought to influence cell adhesion and inhibit cell growth and metastasis [15–17]. In this study, we observed that ALDOB promotes TET1 expression. Knock down of TET1 by siRNA promoted HCC migration and reversed the inhibition of metastasis induced by ALDOB. All of these results suggest that ALDOB inhibits cell metastasis, partly through its regulation of TET1. Moreover, some reports have shown that addition of MG to cultures can elevate TET1 expression in lens epithelial cells [21]. We validated this observation by adding MG to HCC cells. This experiment indicated that elevation of TET1 by ALDOB might occur at least partly through MG. However, as described above, since we lack the means to eliminate MG under experimental conditions, the relationship between ALDOB/MG/TET1is still unclear and requires further investigation.

In conclusion, this study investigated the expression of ALDOB and its value as a prognostic biomarker in HCC. It also explored the biological function of ALDOB in HCC. We observed that the downregulation of ALDOB is a predictor of high-risk metastasis and recurrence in HCC patients. These patients should be closely monitored and should receive appropriate adjuvant therapies for a better prognosis. We also found that ALDOB inhibits HCC metastasis by elevating TET1, a process that may involve MG. However, the detailed mechanism of this process is yet to be elucidated.

## Materials and methods

### Patients and specimens

The study protocol was approved by the clinical research ethics committee of the Eastern Hepatobiliary Surgery Hospital. Written informed consent was obtained from all patients according to the policies of the committee. Any information that could identify the patients was not included in this article.

This study included 29 HCC tissues and paired non-tumorous tissues (including 14 MIH and 15 MAH) for microarray analysis. Another 50 HCC specimens (including 20 MIH with and 30 MAH) were used for the validation of microarray data by qRT-PCR analysis. The MIH and MAH status was defined as described previously [34, 35]. The MIH group was composed of patients with solitary HCC that was accompanied either by portal vein metastasis or by venous metastases at follow-up, or those who had two nodules present at the time of sample collection. The MAH group was restricted to only those patients with solitary HCC and no recurrence at follow-up. Samples from 25 patients with HCC and portal vein tumor thrombosis (PVTT) were used to test the expression level of ALDOB in paired HCC/non-tumor tissues/PVTT by qRT-PCR and IHC. Another 288 HCC cases and paired non-tumor tissues were used to validate ALDOB protein level by IHC. Moreover, a large HCC cohort of 313 patients with HCC (randomly collected from January 2006 to September 2010) who received a high-quality follow-up, were used to examine ALDOB protein levels and the clinical significance of ALDOB by tissue microarray (TMA) and IHC. Normal liver tissues were collected from patients who underwent resection of hepatic hemangiomas. All liver specimens were randomly selected from patients who underwent curative hepatectomy, and all specimens were pathologically confirmed at the Eastern Hepatobiliary Surgery Hospital between 2005 and 2010 [36].

The preoperative diagnosis was obtained as described previously, and surgical procedures were performed on the patients with HCC as described previously [37]. The clinical characteristics of the HCC cohort are listed in Additional file 2: Table S1. The differentiation of HCC was defined according to the criteria proposed by Edmondson and Steiner (I, well-differentiated; II, moderately differentiated; III, poorly differentiated; IV, undifferentiated). Micro-metastases were defined as tumors that were located adjacent to the border of the primary tumor and that could only be observed under the microscope. Tumor staging was conducted according to the BCLC staging system and the sixth edition of the Tumor-Node-Metastasis (TNM) classification system published by the International Union Against Cancer.

All HCC specimens were obtained immediately after hepatectomy. Tissues were then fixed in 10 % buffered formalin and embedded in paraffin. Fresh specimens used in this study were snap-frozen from tissues prior to formalin fixation and were then transferred to liquid nitrogen and stored at −80 °C until use.

### Follow-up

All patients with HCC received check-ups every 2–3 months during the first 2 years and every 3–6 months there after until the follow-up period ended in January 2013. Physicians who were blinded to the study performed the follow-up examinations. Serum AFP levels and abdominal ultrasound examinations were performed every month during the first year after surgery and every 3–6 months thereafter. Computed tomography and/or magnetic resonance imaging were performed every 3–6 months or when a recurrence was suspected. A diagnosis of recurrence was based on preoperative diagnosis criteria. Once recurrence was confirmed, further treatment was implemented based on the tumor diameter, the number of tumors, the location of the tumor, and the extent of vessel invasion as well as liver function and performance statuses. The recurrence-free survival (RFS) was calculated from the date of tumor resection until the detection of tumor recurrence, death from a cause other than HCC, or the last follow-up visit. The overall survival (OS) was defined as the length of time between surgery and either the death of the patient or the last follow-up visit.

### Cell lines

The SMMC7721, LM3, LM6, HepG2, Huh7, and Hep3B cells were obtained from the Chinese Academy of Sciences Cell Bank and were authenticated by the providers by DNA-Fingerprinting analysis or isoenzyme analysis. All cell lines were used at early passages and no morphological changes or altered growth rates were observed during maintenance of the cultures. Cells were grown in Dulbecco’s modified Eagle’s medium with 10 % fetal bovine serum (Gibco BRL). Cells were maintained in an atmosphere of 5 % CO2 in a humidified 37 °C incubator.

### Extraction of RNA, Preparation of cDNA and Quantitative real-time PCR (qRT-PCR)

Total RNA was extracted from snap-frozen tissues with Trizol reagent (Takara, Dalian, China) according to the manufacturer’s instructions. The quality of the total RNA was assessed by a Nanodrop 2000 and agarose gel electrophoresis. First-strand cDNA was synthesized from 1–2 μg of total RNA using random primers and M-MLV Reverse Transcriptase (Invitrogen, CA). Real-time PCR was performed according to the Taqman probe assay protocol or the SYBR Green protocol in a StepOne Plus system (Applied Biosystems, Foster City, CA) with β-actin as the endogenous control. The relative mRNA levels were calculated based on the Ct values and were according to the β-actin expression. The primer and probe sequences are listed in Additional file 2: Table S3.

### Western blot analysis

Total protein was extracted from snap-frozen tissues with RIPA Lysis Buffer and PMSF (Beyotime Co., China) according to the manufacturer’s instructions. Western blotting was performed as described previously [38]. Antibody dilutions were 1:1500 for the ALDOB polyclonal antibody (Proteintech Group, Inc., Chicago, USA) and 1:5000 for β-actin (Sigma-Aldrich, USA). Antibody binding was detected with an Odyssey infrared scanner (Li-Cor Biosciences Inc).

### Tissue microarray construction and immunohistochemical analysis

The tissue microarray (TMA) was constructed as described previously [39]. Briefly, all samples from patients in the HCC cohort were reviewed histologically by hematoxylin and eosin staining. Representative areas were premarked on the paraffin blocks, away from necrotic and hemorrhagic tissue. Two 1.0-mmcores were extracted from each tumor, were paired with non-tumor tissue and were mounted on a new recipient block with a semi-automated arraying device (TMArrayer, Pathology Devices, Westminster, MD, USA).

Immunohistochemistry was performed on the TMA with a two-step immunoperoxidase technique [40]. The ALDOB polyclonal antibody (Proteintech Group, Inc, Chicago, USA) diluted 1:60 was used as the primary antibody. Briefly, after heating the sections in 10 mmol/L citrate buffer for antigen retrieval, the sections were incubated first with the primary antibody and then with the secondary antibody for an hour at room temperature. Finally, the sections were developed in diaminobenzidine solution under a microscope and counter-stained with hematoxylin.

The immunohistochemical stains were assessed by three separate observers who had no knowledge of patient characteristics. ALDOB staining was abundant in the cytoplasm and nucleus, which was similar to previous studies [41, 42]. The expression was recorded after an evaluation of the staining intensity of positive cells (0 = none; 1 = +weak; 2 = ++ intermediate, 3 = +++ strong). Scoring differences were discussed until a consensus was reached. A low level of ALDOB in the HCC specimens was defined as staining intensity that was designated as either “none” or “weak.”

### Wound healing migration assay

Wound healing migration assays were performed as described previously [38]. Briefly, 1 × 10^5^ cells were plated in each well of 6-well plates. Once cells were attached, they were scraped to form a wound in the middle of the plates and the medium was replaced with serum-free medium. We measured the area of healing across the line after an incubation of 36 h.

### Transwell assays

Millicell 24-well culture insert plates (Millipore, USA) and polycarbonate membranes with a pore size of 8 μm were used in Transwell assays as described previously [38]. First, The insert plates were equilibrated with 0.5 ml of DMEM for1 h at 37 °C in 5 % CO_2,_ which was then replaced with 0.5 ml of DMEM supplemented with 10 % FBS in the lower chambers. In all, 50,000 cells in 400 μl of serum-free DMEM were loaded into the upper chambers. After 24 h of incubation, the insert plates were rinsed with PBS and the upper surfaces of the membranes were scraped toremove the cells. The cells on the underside of the membrane were stained with Giemsa stain and counted under a microscope. Cells from each culture condition were examined in quadruplicate.

### Animal studies

The animal studies were approved by the Institutional Animal Care and Use Committee of the Second Military Medical University, Shanghai, China. To explore the effects of ALDOB on tumor growth *in vivo*, 1 × 10^7^SMMC7721 cells thatstablyexpressedALDOB (SMMC-ALDOB) and control cells (SMMC-GFP) were subcutaneously implanted into the bilateral armpit of nine BALB/C nude mice. The tumor volume was measured every week after implantation (the volume V = length × width × length × 1/2). All mice were sacrificed five weeks later, and sections of tumor tissues were transplanted to establish an orthotopic model as described previously [37, 43]. A tail vein injection model was also used to evaluate the potential of the cells to metastasize to the lungs. The metastases were monitored by the IVIS@ Lumina II system (Caliper Life Sciences, Hopkinton, MA) for 10 min after intraperitoneal injection of 4.0 mg of luciferin (Gold Biotech) in 50 μl of saline.

### Statistical analysis

All statistical analyses were performed with SPSS version 18.0 software. The *χ*^2^ test or Fisher’s exact test were used to compare qualitative variables, and the Student’s *t*-test or the Mann–Whitney test were used to compare continuous variables. Survival curves were calculated according to the Kaplan–Meier method and were compared by a log-rank test. A Cox proportional hazards model was used to determine the independent factors of survival and recurrence based on variables selected after the univariate analysis. A difference was defined as significant at *P* < 0.05.

## Additional files

Additional file 1: Figure S1. (A) Average expression level of ALDOB in HCC and paired non-tumor tissue by IHC; representative IHC stains of ALDOB areillustrated in (B). (C) Immunohistochemistry score distribution of the tumors of the 313 patients with HCC. (D) Representative images of the immunohistochemistry score of ALDOB. (TIFF 5836 kb) Additional file 2: Table S1-S3. Clinical characteristics of 313 HCC patients according to ALDOB expression. **Table S2.** Univariate analysis of outcomes of 314 patients with HCC. **Table S3.** The list of primers and siRNA sequences used in study. (DOCX 19 kb) Additional file 3: Figure S2. (A) Exact copy number of ALDOB mRNA in 1 ng RNA from HCC cell lines (upper). The ALDOB protein level in HCC cell lines by western blot (lower). (B) Exact copy number of ALDOA mRNA in 1 ng RNA from HCC cell lines (upper). The ALDOA protein level in HCC cell lines by western blot (lower). (C) The ALDOB protein level in ALDOB stably expressing cell lines and in paired controls as measured by western blot. (D) The relative expression of ALODA in ALDOB expressing cell lines compares to controls. The expression levels of the genes were analyzed by RT-PCR and normalized to β-actin. (TIFF 10926 kb) Additional file 4: Figure S3. The scratch wound healing assays and Transwell migration assays showed that the overexpression of ALDOB inhibits cell migratory properties in the HCC cell line LM6 (A-D). The representative results and statistical analysis are shown. Scale bars, 200 μM. (E) The growth curves of SMMC-ALD and its controls in CCK8 assay. (F) Images of the tumors that developed in the mouse models after subcutaneous injection of SMMC-ALDOB and SMMC-GFP cells. (TIFF 6500 kb) Additional file 5: Figure S4. (A) Relative expression of glycolysis genes in SMMC-ALDOB and SMMC-GFP cells. The expression levels of the genes were analyzed by RT-PCR and normalized to β-actin. (B) The relative glucose concentration in SMMC-ALDOB and Huh7-ALDOB cells normalized to that in paired controls under the same conditions. (C) The production of lactic acid in SMMC-ALDOB and Huh7-ALDOB cells as measured by a lactic acid detection kit (Jian Cheng, NanJing, China) according to the manufacturer’s instructions. (D) The production of methylglyoxal in ALDOB stably expressing cells was measured by a methylglyoxal detection kit (JianCheng, NanJing, China; also TSZELISA, Lexington, USA) according to the manufacturer’s instructions after 1:10^5^ dilution. (E) The expression of TET1 in cells cultured in elevated concentrations of MG (0/0.312/0.625/1.25/2.5/5 mM). The expression was analyzed by RT-PCR and was normalized to β-actin. (F) The expression of HOXA9 in ALDOB stably expressing cells and in paired controls. The expression was analyzed by RT-PCR and was normalized to β-actin. (TIFF 2168 kb) Additional file 6: Figure S5. (A&B) The GO biological process analysis of up-regulated genes (A) and down-regulated genes (B) in SMMC-ALDOB cells compared with controls. (C&D) The Parthway analysis of up-regulated genes (C) and down-regulated genes (D) in SMMC-ALDOB cells compared with controls. (TIFF 670 kb)

## Abbreviations

ALDOB: Aldolase B
HCC: Hepatocellular carcinoma
TET1: Ten–eleven translocation 1
RFS: Recurrence free survival
OS: Overall survival
