# Supplementary figures and images for: Aldolase B inhibits metastasis through Ten–Eleven Translocation 1 and serves as a prognostic biomarker in hepatocellular carcinoma
Source: Mol Cancer. 2015 Sep 17;14:170. doi: 10.1186/s12943-015-0437-7 (PMC4574028; doi:10.1186/s12943-015-0437-7)

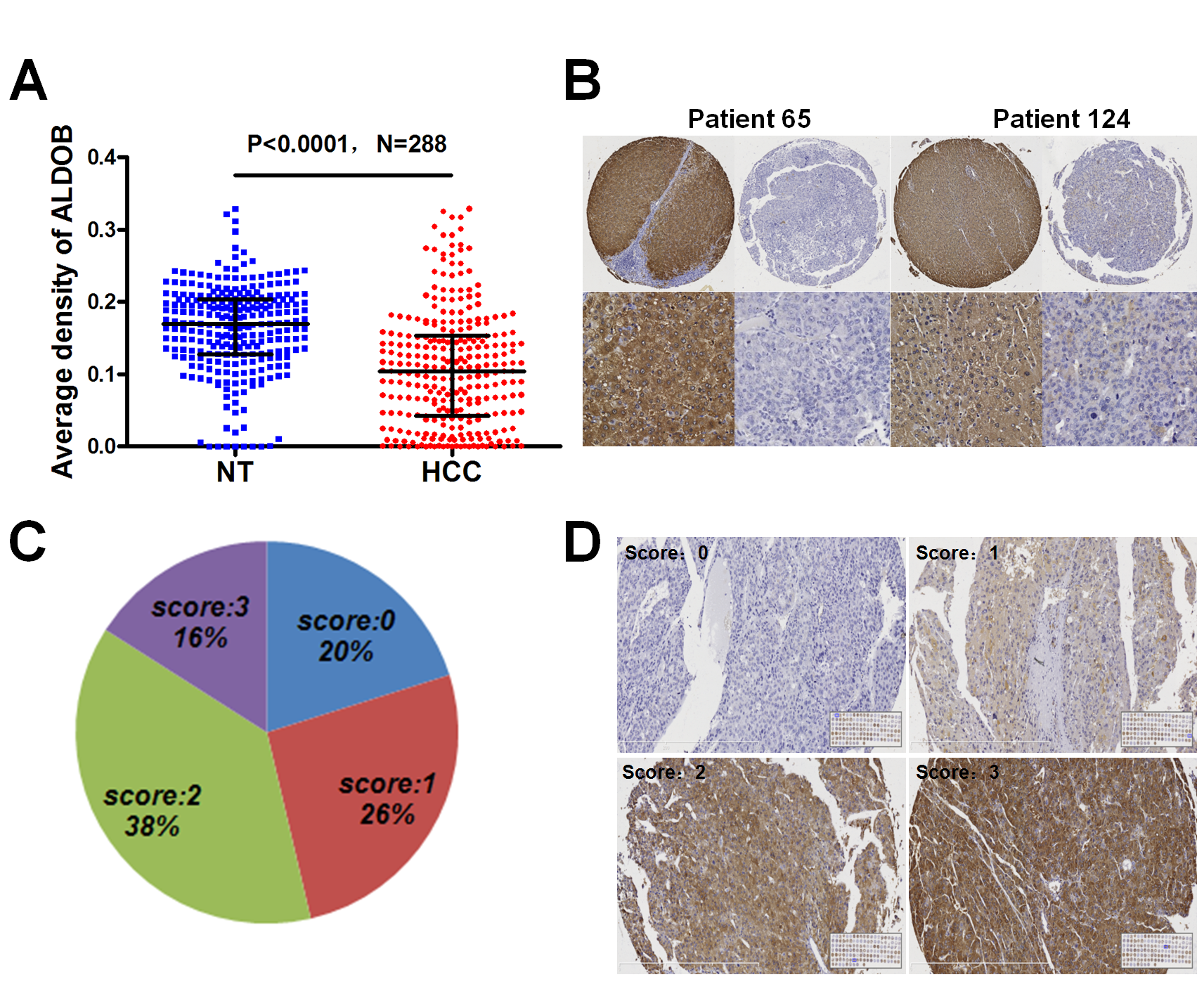

Supplement: Additional file 1: Figure S1. — (A) Average expression level of ALDOB in HCC and paired non-tumor tissue by IHC; representative IHC stains of ALDOB areillustrated in (B). (C) Immunohistochemistry score distribution of the tumors of the 313 patients with HCC. (D) Representative images of the immunohistochemistry score of ALDOB. (TIFF 5836 kb) [file 12943_2015_437_MOESM1_ESM.tif]

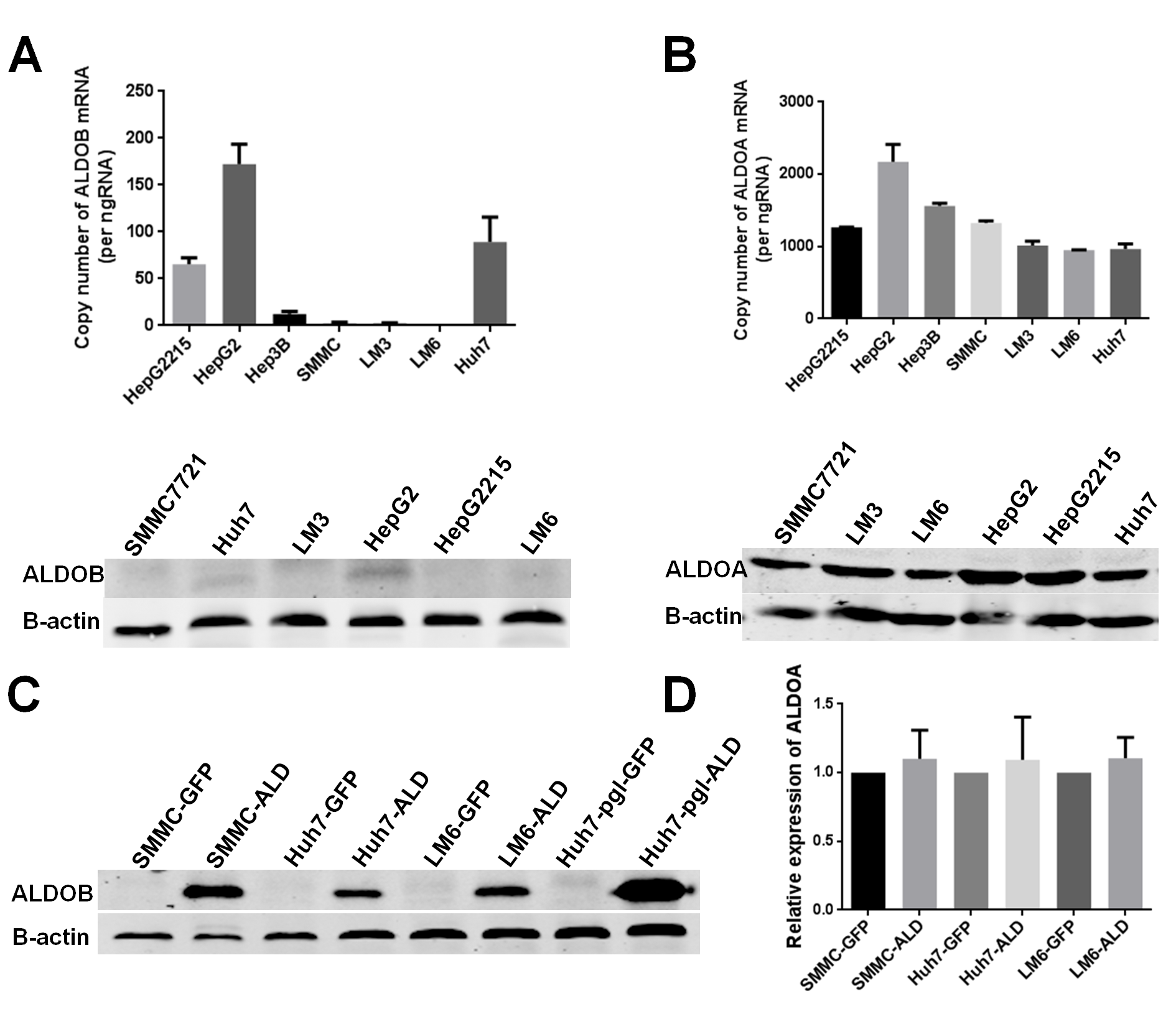

Supplement: Additional file 3: Figure S2. — (A) Exact copy number of ALDOB mRNA in 1 ng RNA from HCC cell lines (upper). The ALDOB protein level in HCC cell lines by western blot (lower). (B) Exact copy number of ALDOA mRNA in 1 ng RNA from HCC cell lines (upper). The ALDOA protein level in HCC cell lines by western blot (lower). (C) The ALDOB protein level in ALDOB stably expressing cell lines and in paired controls as measured by western blot. (D) The relative expression of ALODA in ALDOB expressing cell lines compares to controls. The expression levels of the genes were analyzed by RT-PCR and normalized to β-actin. (TIFF 10926 kb) [file 12943_2015_437_MOESM3_ESM.tif]

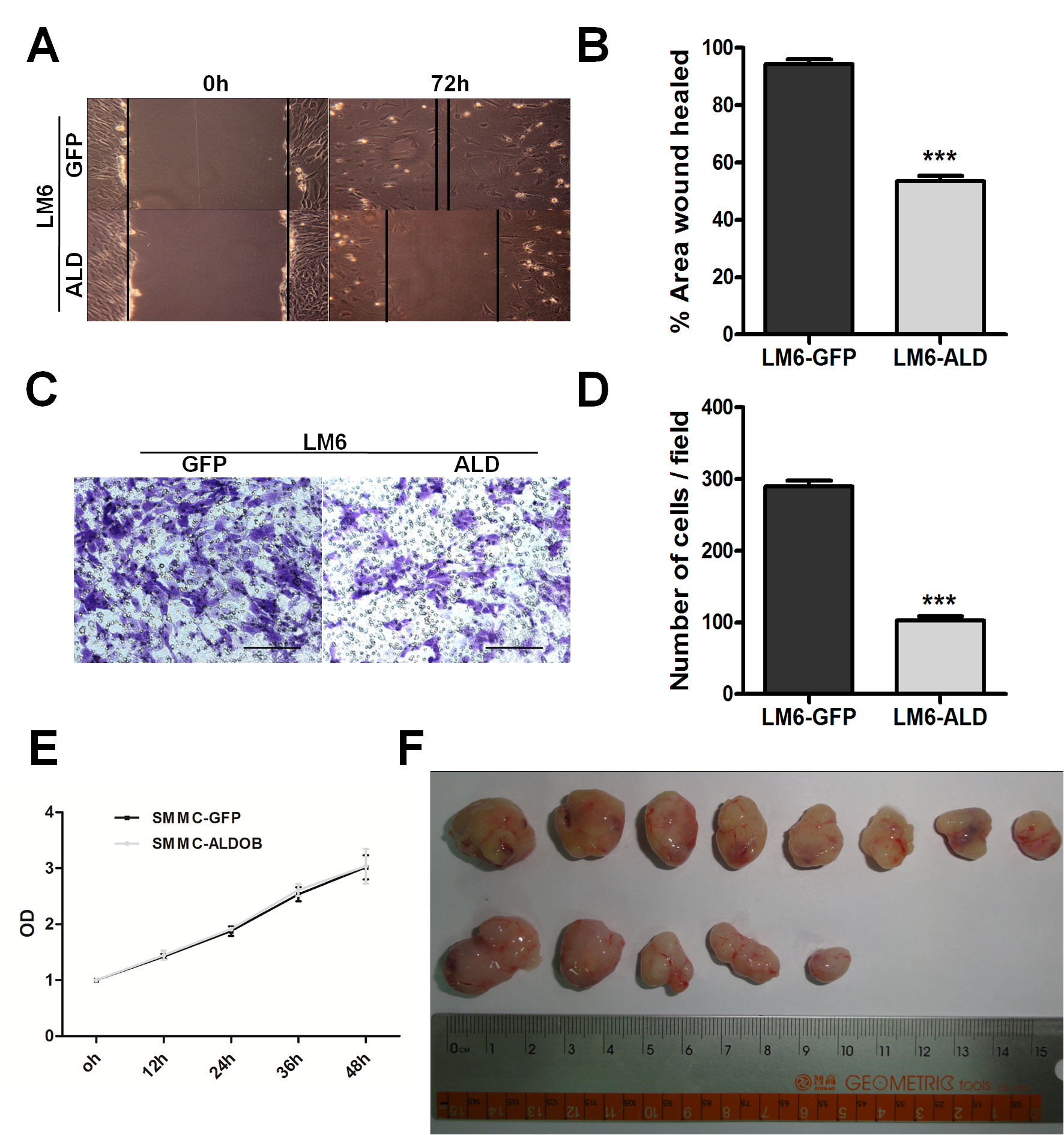

Supplement: Additional file 4: Figure S3. — The scratch wound healing assays and Transwell migration assays showed that the overexpression of ALDOB inhibits cell migratory properties in the HCC cell line LM6 (A-D). The representative results and statistical analysis are shown. Scale bars, 200 μM. (E) The growth curves of SMMC-ALD and its controls in CCK8 assay. (F) Images of the tumors that developed in the mouse models after subcutaneous injection of SMMC-ALDOB and SMMC-GFP cells. (TIFF 6500 kb) [file 12943_2015_437_MOESM4_ESM.tif]

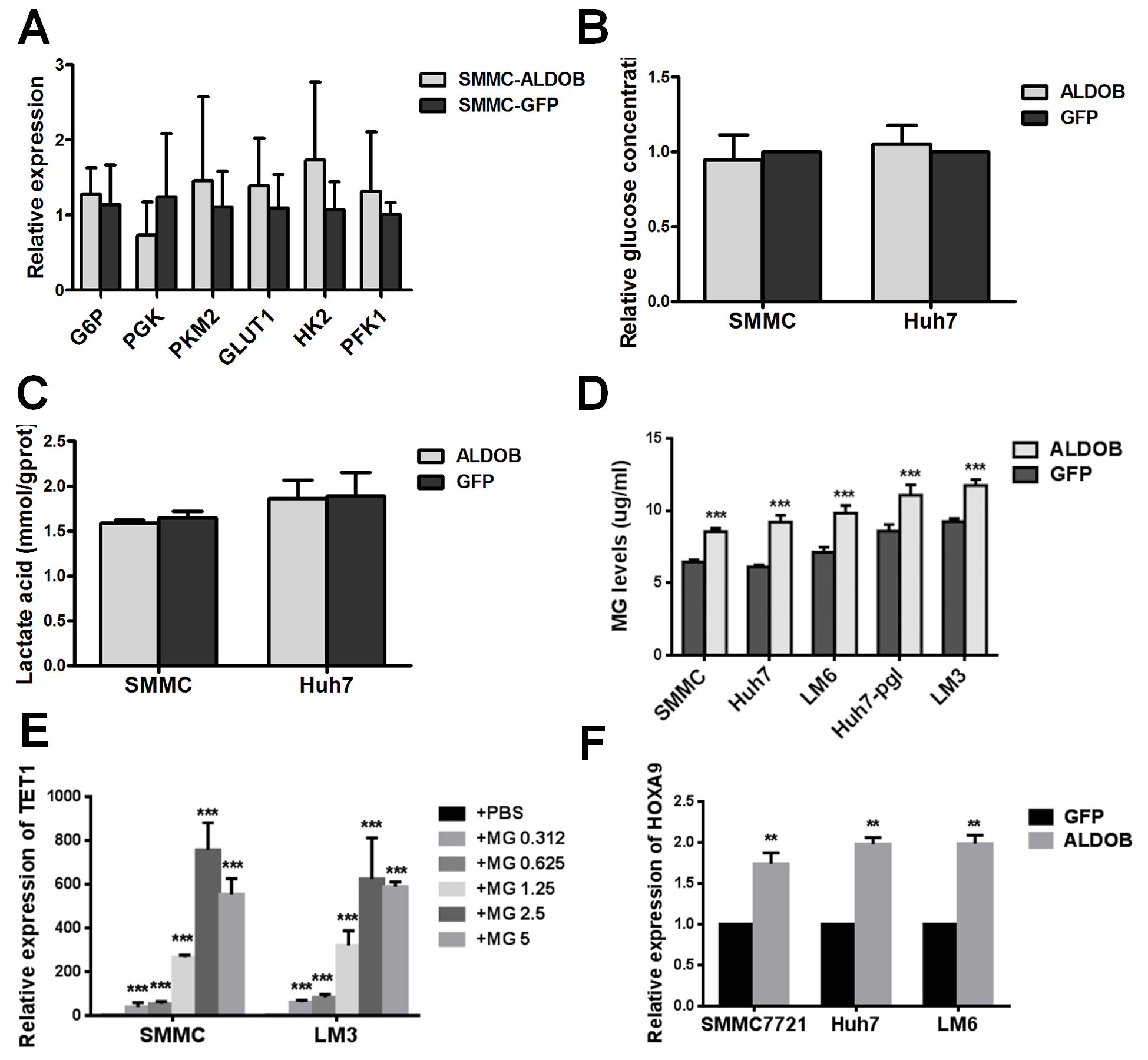

Supplement: Additional file 5: Figure S4. — (A) Relative expression of glycolysis genes in SMMC-ALDOB and SMMC-GFP cells. The expression levels of the genes were analyzed by RT-PCR and normalized to β-actin. (B) The relative glucose concentration in SMMC-ALDOB and Huh7-ALDOB cells normalized to that in paired controls under the same conditions. (C) The production of lactic acid in SMMC-ALDOB and Huh7-ALDOB cells as measured by a lactic acid detection kit (Jian Cheng, NanJing, China) according to the manufacturer’s instructions. (D) The production of methylglyoxal in ALDOB stably expressing cells was measured by a methylglyoxal detection kit (JianCheng, NanJing, China; also TSZELISA, Lexington, USA) according to the manufacturer’s instructions after 1:105 dilution. (E) The expression of TET1 in cells cultured in elevated concentrations of MG (0/0.312/0.625/1.25/2.5/5 mM). The expression was analyzed by RT-PCR and was normalized to β-actin. (F) The expression of HOXA9 in ALDOB stably expressing cells and in paired controls. The expression was analyzed by RT-PCR and was normalized to β-actin. (TIFF 2168 kb) [file 12943_2015_437_MOESM5_ESM.tif]

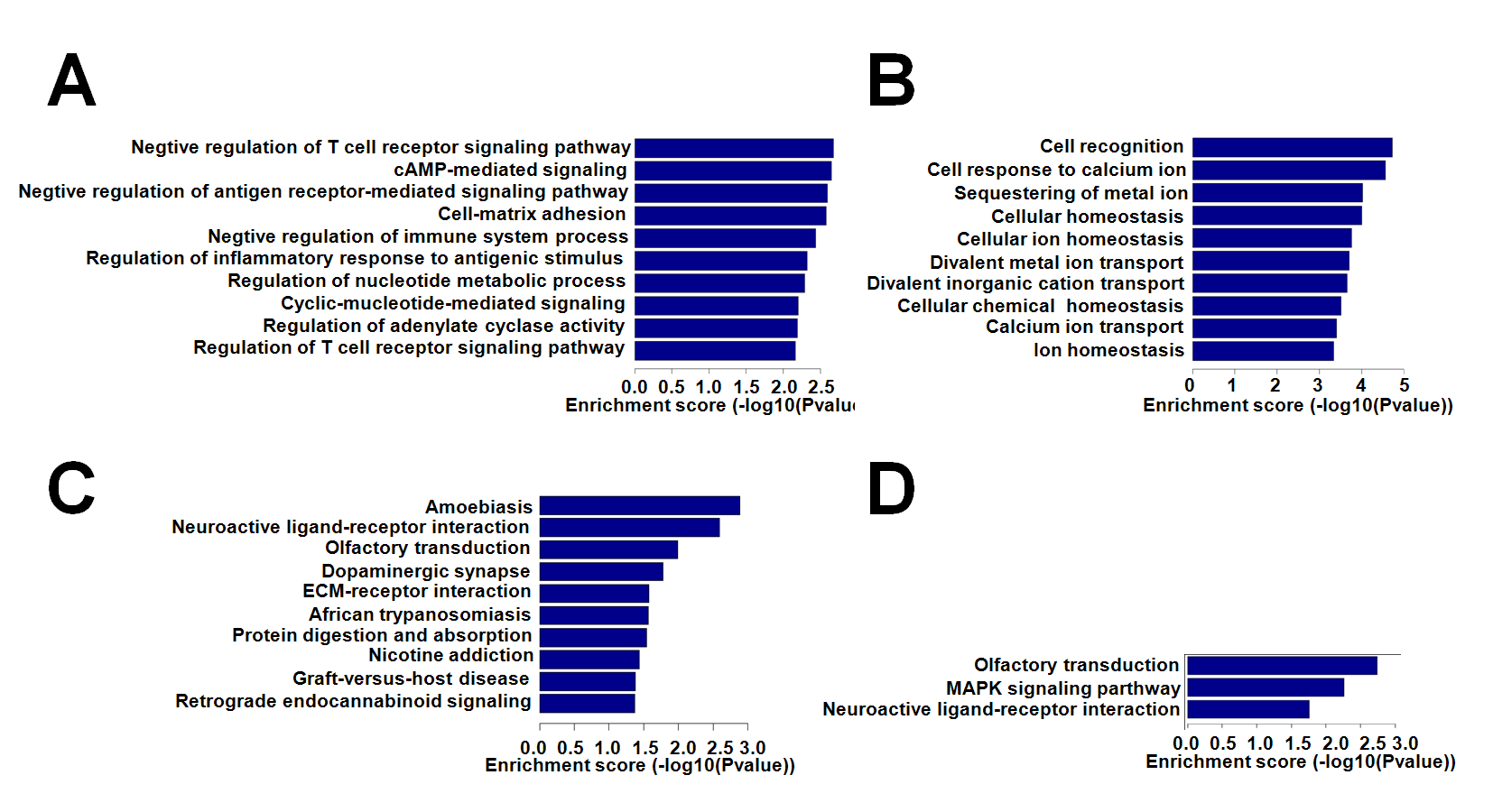

Supplement: Additional file 6: Figure S5. — (A&B) The GO biological process analysis of up-regulated genes (A) and down-regulated genes (B) in SMMC-ALDOB cells compared with controls. (C&D) The Parthway analysis of up-regulated genes (C) and down-regulated genes (D) in SMMC-ALDOB cells compared with controls. (TIFF 670 kb) [file 12943_2015_437_MOESM6_ESM.tif]
