# Supplementary material for: Aldolase B inhibits metastasis through Ten–Eleven Translocation 1 and serves as a prognostic biomarker in hepatocellular carcinoma
Source: Mol Cancer. 2015 Sep 17;14:170. doi: 10.1186/s12943-015-0437-7 (PMC4574028; doi:10.1186/s12943-015-0437-7)
Supplement: Additional file 2: Table S1-S3. — Clinical characteristics of 313 HCC patients according to ALDOB expression. Table S2. Univariate analysis of outcomes of 314 patients with HCC. Table S3. The list of primers and siRNA sequences used in study. (DOCX 19 kb) [file 12943_2015_437_MOESM2_ESM.docx]

**Table S1**. **Clinical characteristics of 313 HCC patients according to ALDOB expression.**

| **Variable** | **ALDOB expression^▲^** | | **P-value** |
| --- | --- | --- | --- |
|  | **High** | **Low** |  |
| **All cases** | 168 | 145 |  |
| **Age(year),>55: ≤55** | 60:108 | 47:98 | 0.539 |
| **Gender, male:female** | 155:13 | 121:24 | 0.016^★^ |
| **HBs antigen**  **positive:negative** | 143:25 | 129:16 | 0.315 |
| **HBe antigen**  **positive:negative** | 37:131 | 28:117 | 0.555 |
| **Liver cirrhosis,**  **Present: Absent** | 106:62 | 99:46 | 0.336 |
| **Liver function,**  **Child A:Child B** | 17:151 | 10:135 | 0.311 |
| **AFP(ug/L),>20:≤20** | 99:69 | 98:47 | 0.114 |
| **Tumor size(cm),>5:≤5** | 71:97 | 84:61 | 0.006^★^ |
| **No. tumor,**  **Solitary: Multiple** | 136:32 | 114:31 | 0.608 |
| **Differentiation, I+II:III+IV** | 22:146 | 12:133 | 0.172 |
| **Micro-vascular invasion,**  **Present: Absent** | 93:75 | 92:53 | 0.147 |
| **Macro-vascular invasion,**  **Present: Absent** | 14:154 | 15:130 | 0.541 |
| **Micro-metastases,**  **Present: Absent** | 123:45 | 107:38 | 0.908 |
| **Encapsulation,**  **Present: Absent** | 78:90 | 42:103 | 0.002^★^ |
| **TNM stage, I: II+III** | 57:111 | 46:99 | 0.679 |
| **BCLC stage, 0-A:B-C** | 132:36 | 105:40 | 0.205 |
| **Early recurrence,**  **Present: Absent** | 47:121 | 65:80 | 0.002^★^ |

**Data are expressed as ratios.**

**▲ALDOB downexpression was defined as staining intensity in HCC weaker than paired non-tumorous tissue.**

**★P<0.05 by χ2 test.**

**Table S2.** **Univariate analysis of outcomes of 314 patients with HCC.**

| **Variable** | **Recurrence-Free**  **Survival(%)^▲^** | **P-value** | **Overall Survival(%)^▲^** | **P-value** |
| --- | --- | --- | --- | --- |
| **Age(year),>55: ≤55** | 40.101:40.155 | 0.669 | 57.948:51.597 | 0.978 |
| **Gender, male:female** | 40.536:35.919 | 0.762 | 57.979:43.865 | 0.352 |
| **HBs antigen**  **positive:negative** | 37.996:52.293 | 0.004^★^ | 55.219:57.732 | 0.014^★^ |
| **HBe antigen**  **positive:negative** | 28.582:42.821 | 0.003^★^ | 40.910:59.578 | 0.015^★^ |
| **Liver cirrhosis,**  **Present: Absent** | 36.634:47.350 | 0.006^★^ | 55.081:53.810 | 0.133 |
| **Liver function,**  **Child A:Child B** | 41.095:31.148 | 0.146 | 58.289:42.000 | 0.118 |
| **AFP(ug/L),>20:≤20** | 37.323:45.301 | 0.063 | 48.566:61.302 | 0.239 |
| **Tumor size(cm),>5:≤5** | 33.821:46.569 | 0.002^★^ | 43.376:65.761 | 0.000^★^ |
| **No. tumor,**  **Solitary: Multiple** | 42.079:33.018 | 0.057 | 59.621:42.513 | 0.019^★^ |
| **Differentiation, I+II:III+IV** | 51.434:38.927 | 0.050 | 56.853:56.366 | 0.256 |
| **Micro-vascular invasion,**  **Present: Absent** | 34.886:48.156 | 0.001^★^ | 51.848:59.206 | 0.005^★^ |
| **Macro-vascular invasion,**  **Present: Absent** | 25.103:41.700 | 0.014^★^ | 30.414:59.380 | 0.000^★^ |
| **Micro-metastases,**  **Present: Absent** | 39.462:42.698 | 0.415 | 50.654:56.875 | 0.879 |
| **Encapsulation,**  **Present: Absent** | 49.301:34.694 | 0.001^★^ | 59.926:51.869 | 0.001^★^ |
| **TNM stage, I: II-III** | 49.227:35.981 | 0.002^★^ | 60.208:52.871 | 0.005^★^ |
| **BCLC stage, 0-A:B-C** | 44.106:28.241 | 0.000^★^ | 61.789:37.872 | 0.000^★^ |
| **ALDOB expression**  **High:Low** | 45.868: 32.519 | 0.003^★^ | 57.426:50.135 | 0.002^★^ |

**▲The time follow-up ended is used to calculate the Recurrence-Free Survival and Overall Survival**

**★P<0.05 by Long-rank test.**

**Table S3. The list of primers and siRNA sequences used in study.**

| **name** | **sequeces** |
| --- | --- |
| **ALDOB qPCR primer** | **Forward:** **GCTATCCAGGAAAACGCCAACGC** |
|  | **Reverse:** **TTCACTCATGCCACCAGACAAAA** |
| **β-actin qPCR primer** | **Forward:** **GCTGGTGACAGGGAAGAC** |
|  | **Reverse:** **GGGATGGTGGGTGTAAGA** |
| **TET1 qPCR primer** | **Forward:** **CTTAGGGAGTAACACTGAGACCGT** |
|  | **Reverse:** **GGAGAAGCCTGGAGATGCC** |
| **SiTET1** | **Sense:** **CCTTGATAGAATCACTCAGTT** |
|  | **Antisense: CUGAGUGAUUCUAUCAAGGTT** |
| **SiALDOB** | **Sense:** **GAAGUAUACUCCAGAACAATT** |
|  | **Antisense:** **UUGUUCUGGAGUAUACUUCTT** |
|  |  |
